# Application of MRI image segmentation algorithm for brain tumors based on improved YOLO

**DOI:** 10.3389/fnins.2024.1510175

**Published:** 2025-01-07

**Authors:** Tao Yang, Xueqi Lu, Lanlan Yang, Miyang Yang, Jinghui Chen, Hongjia Zhao

**Affiliations:** ^1^The First Clinical Medical College, The Affiliated People’s Hospital of Fujian University of Traditional Chinese Medicine, Fuzhou, Fujian, China; ^2^School of Biomedical Engineering, Southern Medical University, Guangzhou, China; ^3^The Affiliated People’s Hospital of Fujian University of Traditional Chinese Medicine, Fuzhou, China

**Keywords:** artificial intelligence, image segmentation, brain tumor, magnetic resonance, YOLOv5s

## Abstract

**Objective:**

To assist in the rapid clinical identification of brain tumor types while achieving segmentation detection, this study investigates the feasibility of applying the deep learning YOLOv5s algorithm model to the segmentation of brain tumor magnetic resonance images and optimizes and upgrades it on this basis.

**Methods:**

The research institute utilized two public datasets of meningioma and glioma magnetic resonance imaging from Kaggle. Dataset 1 contains a total of 3,223 images, and Dataset 2 contains 216 images. From Dataset 1, we randomly selected 3,000 images and used the Labelimg tool to annotate the cancerous regions within the images. These images were then divided into training and validation sets in a 7:3 ratio. The remaining 223 images, along with Dataset 2, were ultimately used as the internal test set and external test set, respectively, to evaluate the model’s segmentation effect. A series of optimizations were made to the original YOLOv5 algorithm, introducing the Atrous Spatial Pyramid Pooling (ASPP), Convolutional Block Attention Module (CBAM), Coordinate Attention (CA) for structural improvement, resulting in several optimized versions, namely YOLOv5s-ASPP, YOLOv5s-CBAM, YOLOv5s-CA, YOLOv5s-ASPP-CBAM, and YOLOv5s-ASPP-CA. The training and validation sets were input into the original YOLOv5s model, five optimized models, and the YOLOv8s model for 100 rounds of iterative training. The best weight file of the model with the best evaluation index in the six trained models was used for the final test of the test set.

**Results:**

After iterative training, the seven models can segment and recognize brain tumor magnetic resonance images. Their precision rates on the validation set are 92.5, 93.5, 91.2, 91.8, 89.6, 90.8, and 93.1%, respectively. The corresponding recall rates are 84, 85.3, 85.4, 84.7, 87.3, 85.4, and 91.9%. The best weight file of the model with the best evaluation index among the six trained models was tested on the test set, and the improved model significantly enhanced the image segmentation ability compared to the original model.

**Conclusion:**

Compared with the original YOLOv5s model, among the five improved models, the improved YOLOv5s-ASPP model significantly enhanced the segmentation ability of brain tumor magnetic resonance images, which is helpful in assisting clinical diagnosis and treatment planning.

## Introduction

Brain tumors pose a significant threat to human health, and their high mortality rate makes them one of the focal points of medical research (Liu et al., 2020). Epidemiological data indicates that brain tumors account for 1.5% of all cancer incidences, yet they cause a mortality rate as high as 3% (Fitzmaurice et al., 2019). Diagnostic modalities for brain tumors include Computed Tomography (CT) and Magnetic Resonance Imaging (MRI), with MRI’s superior soft tissue contrast becoming a critical diagnostic tool. In clinical settings, physicians rely on accurate differentiation between tumor and normal tissues in imaging to ascertain tumor type, which subsequently informs decisions on surgical margins, radiation therapy, and chemotherapy. This information is also crucial for prognosis assessment and for enhancing patient quality of life.

The diagnosis and treatment of brain tumors have encountered escalating challenges. Traditional imaging techniques, despite advancements in tumor localization and qualitative analysis, are limited in their ability to precisely define tumor margins, analyze tumor heterogeneity, and monitor dynamic changes accurately. Manual segmentation techniques, while intuitive and to some extent meeting clinical needs, are time-consuming and labor-intensive, with a strong subjective nature, which makes it difficult to adapt to the modern medical system’s dual requirements for efficiency and accuracy. Therefore, there is still a need to explore highly automated and precise tumor segmentation techniques.

The relentless progress in Artificial Intelligence (AI) technology has seen AI-assisted diagnosis become an integral part of medical imaging and gradually transition into clinical diagnostics. Intelligent solutions that integrate deep learning with computer vision can markedly increase the level of automation in image analysis, enhancing diagnostic efficiency and objectivity. This technological advancement, by automating the segmentation of tumor regions, reduces the workload on physicians and aids in the precise formulation of treatment plans, leading to more timely and effective patient care.

In the field of deep learning for brain tumor segmentation, Zhao et al. (2016) and his team proposed the Pyramid Scene Parsing Network (PSPNet). This method utilizes a pyramid pooling module to transform the image segmentation challenge into an effective integration of features across different scales. This innovation significantly enhances the model’s dual capabilities of understanding the overall scene and capturing details, achieving comprehensive capture of both global and local features. Ronneberger (2017) designed an encoder-decoder architecture known as U-Net. This framework uses an encoder to deeply extract features from the input image, ensuring the acquisition of rich contextual information. Subsequently, through the decoder phase, the original resolution of the image is restored, and each pixel point is finely classified, achieving high-precision image segmentation tasks. The model design is simple and performs exceptionally well, making it highly suitable for medical image segmentation tasks and has become the most widely used foundational model in the field. Zhang et al. (2020) innovatively introduced the Attention Gate Residual U-Net model (AGResUNet), which, by employing an attention gating mechanism, effectively filters and suppresses irrelevant or noisy feature information related to the task, while significantly enhancing the expression of features closely related to the tumor area. This leads to more accurate identification and positioning in tumor segmentation tasks.

In summary, research on tumor segmentation based on Convolutional Neural Networks (CNN), U-Net, Mask R-CNN, and other methods has yielded many results. Although these methods can effectively perform image segmentation, they require a large amount of annotated data for training and have the disadvantages of high model complexity and significant computational resource consumption. Since the tumor image segmentation task in actual clinical work is carried out in real-time, it is still necessary to explore more efficient and accurate algorithms. In 2016, researchers innovatively proposed a one-stage object detection algorithm, naming it You Only Look Once (YOLO) (Redmon et al., 2016). This framework uses a single neural network as its core architecture, achieving end-to-end object detection tasks. Compared to traditional image segmentation models, this model can directly and accurately predict the coordinates and object positions of the input image, demonstrating high versatility and transferability (Kothai et al., 2024). Among them, You Only Look Once version 5 small (YOLOv5s) is the most lightweight version of the algorithms to date. This algorithm can significantly reduce the model’s complexity and computational costs while maintaining high detection accuracy, and it has been applied to many industrial or agricultural scenarios (Zhang et al., 2024). However, this lightweight design comes at the cost of sacrificing segmentation accuracy to some extent. Especially when performing precise segmentation of small targets or targets in complex backgrounds, it shows limitations (Pei et al., 2022). In clinical practice, the segmentation of brain tumors demands high precision to minimize misdiagnoses and oversights, optimize treatment strategies, and enhance patient outcomes. Consequently, to bolster the segmentation accuracy of the model, it is imperative to implement enhancements to the original YOLOv5 model.

This study focuses on the specific application of YOLOv5 in brain tumor image segmentation, improves the original YOLOv5 model, and discusses the performance of the improved model in brain tumor image analysis, as well as its applicability and limitations in actual clinical scenarios.

The main contributions of this study can be summarized as follows:

1. Improved YOLOv5s Algorithm Model and Multi-scale Feature Capture: We introduce an advanced YOLOv5s algorithm model that incorporates Atrous Spatial Pyramid Pooling (ASPP) and dual attention mechanisms, namely Convolutional Block Attention Module (CBAM) and Coordinate Attention (CA). This integration is pivotal for capturing multi-scale contextual information and sharpening the model’s focus on the critical features within brain tumor regions. By addressing the limitations of traditional models in segmenting tumors of diverse sizes and morphologies, this structured enhancement markedly improves the model’s accuracy and performance in segmenting brain tumor MRI images.
2. Lightweight Design and Performance Balancing: Preserving the high detection velocity inherent to YOLOv5s, we have substantially improved segmentation accuracy through structural refinements. This optimization achieves a commendable equilibrium between the model’s lightweight design and its segmentation precision, catering to the demand for high-precision segmentation in environments with constrained resources.
3. Comprehensive Model Evaluation: The model’s performance is rigorously evaluated using a suite of metrics, including precision, recall, mAP@50, and ablation studies. The findings indicate that the refined model substantially surpasses the original in terms of brain tumor segmentation capabilities. Furthermore, the introduction of the GFLOPs metric quantifies the computational expenditure, elucidating the computational resources required following the enhancement of segmentation capabilities across various improved models.

**Figure 1:**
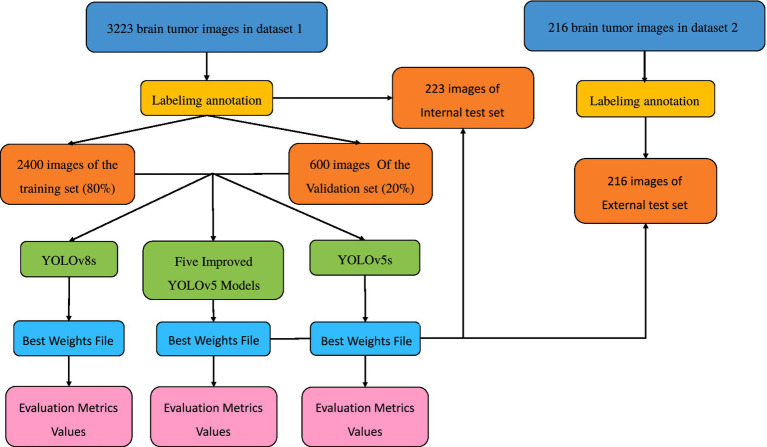
Technical roadmap.

The remainder of this paper is structured as follows: Section 1 outlines the model structures and the innovative improvements of this study. Section 2 describes the model training methods and evaluation metrics. Section 3 discusses the results and facilitates further analysis. Finally, Section 4 discusses the experimental results, draws conclusions, and outlines future work. The overall technical roadmap of this study is shown in Figure 1.

## Improved method

### Original YOLO model

**Figure 2:**
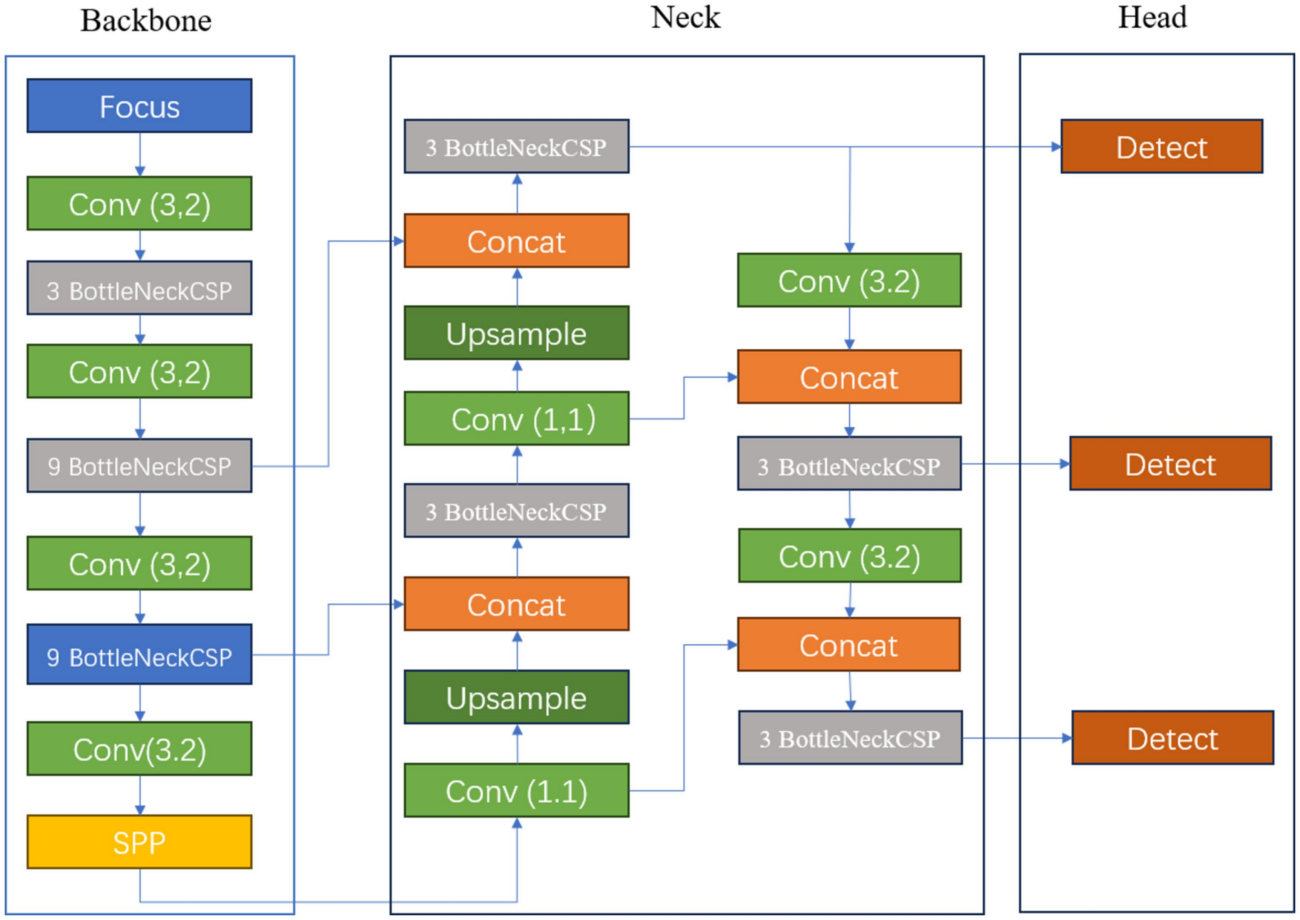
Schematic diagram of YOLOv5s network structure.

Figure 2 illustrates the schematic diagram of the YOLOv5s network structure. The key features of this prediction model’s architecture are as follows: 1. The core feature extraction network of YOLOv5s is based on CSPDarknet53 and incorporates the Cross Stage Partial (CSP) module, a design that reduces computational load and accelerates prediction speed (Zhang et al., 2024). 2. To enhance the detection performance for objects of varying scales, the model employs a path aggregation strategy based on PANet, optimizing the flow of information in the feature pyramid and strengthening the recognition capabilities for multi-scale targets. 3. At the initial stage of model construction, a set of anchor boxes with various sizes and aspect ratios was predefined to account for the diversity of targets in the training data, aiming to achieve more precise target box matching and positional regression in subsequent predictions. 4. Additionally, YOLOv5s utilizes a combination of multi-element loss functions, including classification, bounding box localization, and confidence loss. By synergistically optimizing these loss functions, the accuracy of object detection is further improved. 5. In terms of activation functions, the model opts for Leaky ReLU, a choice that helps mitigate the vanishing gradient problem in deep networks, accelerates model convergence, and enhances training efficiency and the model’s generalization performance. These structural advantages enable YOLOv5s to provide accurate detection results while maintaining high detection speed, making it suitable for real-time applications and resource-constrained environments (see Figure 2).

### Improved YOLO algorithm

#### Improve and innovate ideas

The original YOLOv5s model, while lightweight, inherently compromises segmentation accuracy, necessitating further refinements to bolster its precision in this regard. Dong et al. (2024) enhanced the YOLOv5 model by integrating the CBAM attention mechanism and demonstrated through comparative experiments that the enhanced model surpasses the baseline in detection performance.

However, in the realm of deep learning, a plethora of modules exists, each with unique functionalities: spatial pyramid structures for capturing multi-scale information, pooling mechanisms to refine model accuracy, and attention modules such as Convolutional Block Attention Module (CBAM), Coordinate Attention (CA), and Squeeze-and-Excitation Networks (SE) that direct the model’s focus towards salient features. The efficacy of these modules on model accuracy varies due to their distinct operational mechanisms, underscoring the importance of experimentally integrating or combining them to assess their impact on performance.

This study aims to improve and supplement the deficiencies in the research conducted by Dong and his team. We will focus on two key aspects: the incorporation of Atrous Spatial Pyramid Pooling (ASPP) and attention mechanisms, namely CBAM and CA. The ASPP’s integration aims to enhance the model’s capability to segment tumors of diverse sizes and morphologies by effectively capturing multi-scale contextual information, thereby improving segmentation accuracy. Simultaneously, the inclusion of CBAM and CA will evaluate their potential to augment the model’s recognition and segmentation accuracy of tumor regions. Given that different attention mechanisms may strike varying balances between computational efficiency and segmentation accuracy, comparative experimental analysis will enable the selection of the most apt attention mechanism for specific application scenarios.

#### Improved model architecture

**Figure 3:**
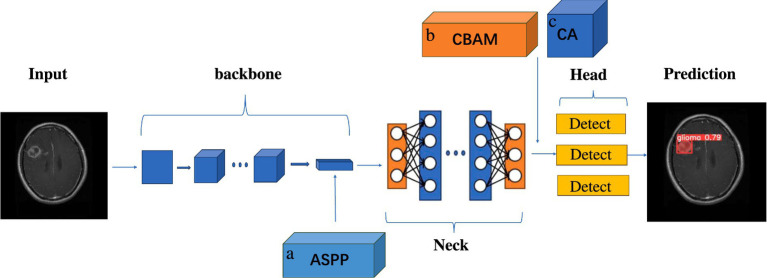
Schematic diagram of the improved YOLOv5s module structure. **A** is ASPP module, replacing SPP structure in the original backbone module with ASPP. **B** is CBAM attention mechanism module. **C** is the CA attention mechanism module, placing the two attention mechanisms between the end of the neck module and the head detection head module.

Figure 3 illustrates the schematic diagram of the improved YOLOv5s model structure. In the course of this investigation, the backbone module of the original model, specifically the Spatial Pyramid Pooling (SPP) structure, was supplanted by the Atrous Spatial Pyramid Pooling (ASPP) mechanism, and two attention mechanisms were integrated between the Neck module and the Head detection module.

To ascertain the efficacy of the amalgamation of Atrous Spatial Pyramid Pooling modules and embedded attention mechanisms in augmenting the performance of the original model, a series of ablation studies were meticulously designed and executed. These studies culminated in the structural refinements and the genesis of several optimized model variants: YOLOv5s-ASPP, YOLOv5s-CBAM, YOLOv5s-CA, YOLOv5s-ASPP-CBAM, and YOLOv5s-ASPP-CA. The ensuing workflow delineates the operational sequence for models that amalgamate Atrous Spatial Pyramid Pooling with attention mechanisms:

1. The input image is subjected to preprocessing protocols, inclusive of resizing and normalization, prior to its ingestion into the YOLOv5 backbone for feature extraction. Upon the extraction of features, the original model’s SPPF module is supplanted by an ASPP module. The ASPP module capitalizes on dilated convolutions to enhance multi-scale feature representation, thereby expanding the receptive field and bolstering the model’s capacity to detect targets across diverse scales.
2. Subsequently, the features extracted by the backbone are sent to the neck for further processing to achieve feature fusion. An attention mechanism is intercalated before each detection head, dynamically modulating the weights accorded to different regions within the feature map. This enhancement sharpens the model’s focus on target-related features, ameliorates its capacity to select and integrate features, and bolsters its ability to handle multi-scale targets while enhancing the utilization of contextual information.
3. Ultimately, the detection head engenders prediction outcomes, which are subjected to non-maximum suppression to eliminate redundant bounding boxes, culminating in the final target segmentation results.

#### Atrous spatial pyramid pooling

**Figure 4:**
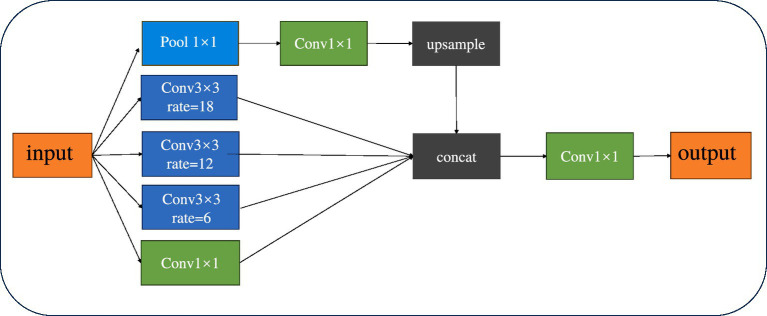
Schematic diagram of ASPP network structure.

Spatial Pyramid Pooling (SPP) is a pooling technique within convolutional neural networks that is used to process inputs of varying sizes. By integrating pooling layers of different scales, it allows for the pooling of input images of any size, thereby enhancing the network’s flexibility and generalization capabilities. Atrous Spatial Pyramid Pooling (ASPP) combines the concepts of SPP and Dilated Convolution (Chen et al., 2017). By incorporating Dilated Convolution, it expands the receptive field of the convolutional kernel without losing resolution. This enables the model to better differentiate between tumor and normal tissues, facilitating precise segmentation even when boundaries are indistinct or sizes are inconsistent. Figure 4 provides a schematic diagram of the ASPP structure.

#### Attention mechanism

##### Convolutional block attention module

**Figure 5:**
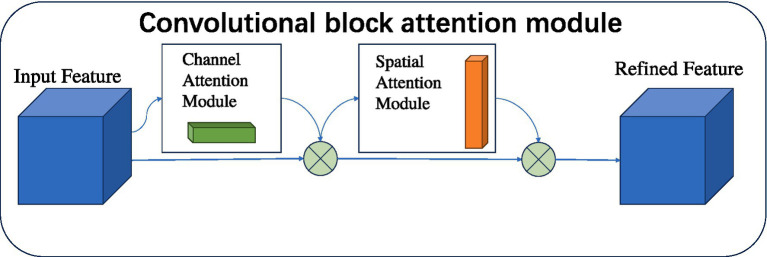
CBAM module structure diagram.

The CBAM attention mechanism is a module designed to enhance the performance of CNNs by allowing the algorithm to focus on more important features extracted (Woo et al., 2018). CBAM combines two submodules, the Channel Attention Module (CAM) and the Spatial Attention Module (SAM), to provide the model with a more comprehensive and effective feature extraction capability. The channel attention mechanism learns the weights of each channel, enabling the model to adaptively adjust the importance of channel features and enhance the modeling capability for different features. The spatial attention module, on the other hand, focuses on the important spatial locations in the feature map. By combining these two, it enhances the model’s attention to key features in the brain tumor region of the image, thereby improving the network performance of the model (Mi et al., 2020; Chen et al., 2020). Figure 5 illustrates the structure of the CBAM attention mechanism.

##### Coordinate attention for efficient mobile network design (CA)

**Figure 6:**
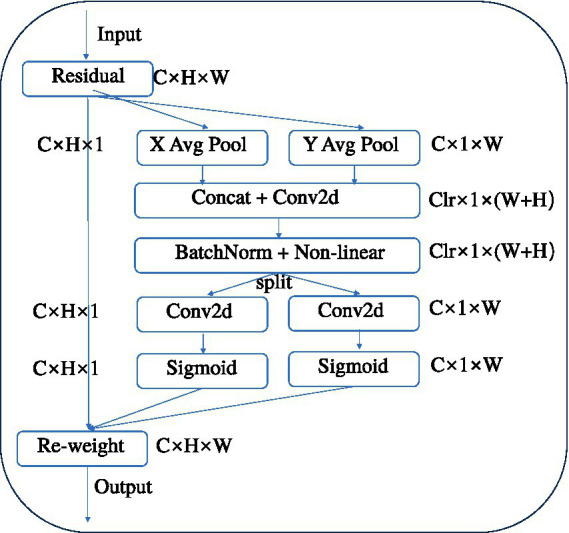
Structure diagram of CA module.

CA is an innovative attention mechanism for mobile networks that enhances the performance of lightweight networks by embedding positional information into channel attention (Hou et al., 2021). It takes into account not only channel information but also direction-related positional information. By integrating convolution with attention, it captures spatial correlations, which helps segmentation models to more accurately locate and identify brain tumor regions in imagery, while being flexible and lightweight enough that the additional computational load is negligible (Xie et al., 2022). Figure 6 is a structural diagram of the CA module.

#### YOLOv8

Given that YOLOv5 and YOLOv8 are designed by the same team and share similar design philosophies, we have decided to include YOLOv8 in our experiments for comparative analysis to explore the performance differences between the two versions in object segmentation tasks. Both employ advanced network structures such as the CSPDarknet backbone and anchor-based detection and segmentation mechanisms, along with non-maximum suppression as a post-processing step, ensuring high efficiency and accuracy.

**Figure 7:**
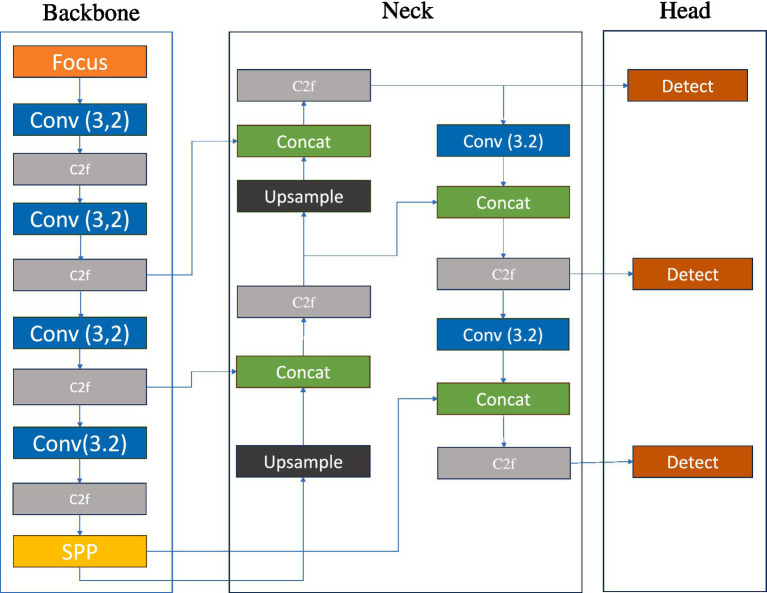
Schematic diagram of YOLOv5s network structure.

However, there are differences in their technical implementations: YOLOv5 is renowned for its efficiency and accuracy, particularly in the detection of small objects, while YOLOv8 builds upon YOLOv5 with further structural optimizations and performance enhancements, introducing innovations such as the SiLU activation function, an improved FPN structure, and task-specific loss functions to adapt to more complex detection and segmentation tasks and to enhance the model’s generalization capabilities (Varghese, 2024). Comparative experiments can help us understand the performance differences between the different versions and provide guidance for model selection and optimization in practical applications. The structure of the YOLOv8 model is shown in Figure 7.

## Materials and methods

### Data acquisition

In this study, brain tumor images were selected from two publicly available datasets on the Kaggle database. The first dataset comprises a total of 3,223 brain tumor MRI images, including 1,581 glioma images and 1,642 meningioma images (Nickparvar, 2021). This dataset is referred to as Dataset 1 and is utilized for model training and for testing model performance with an internal test set. The second dataset contains 101 glioma images and 115 meningioma images, named as Dataset 2 (Bhuvaji et al., 2020), and is used for external testing to evaluate model performance.

**Figure 8:**
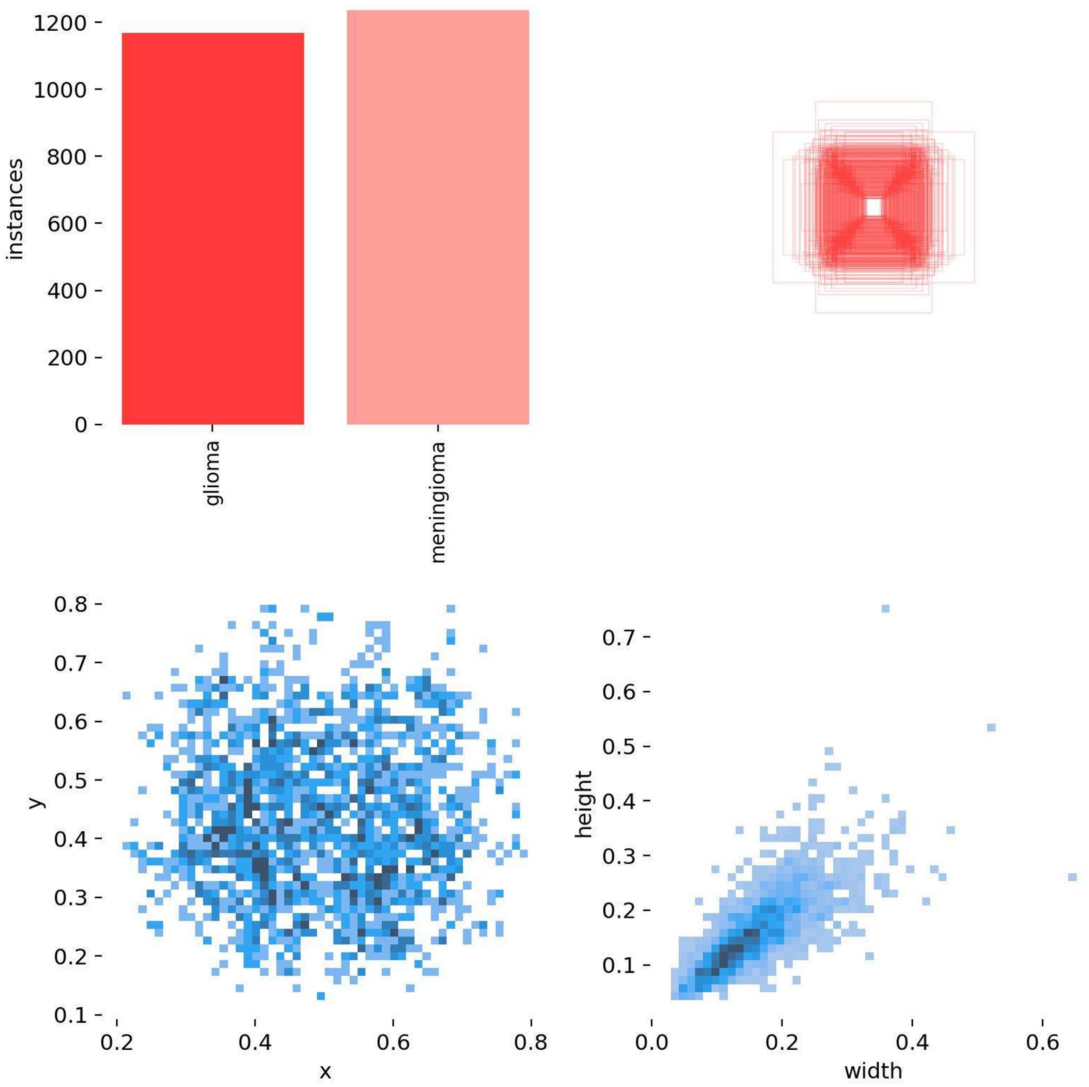
Annotation status in the dataset.

**Figure 9:**
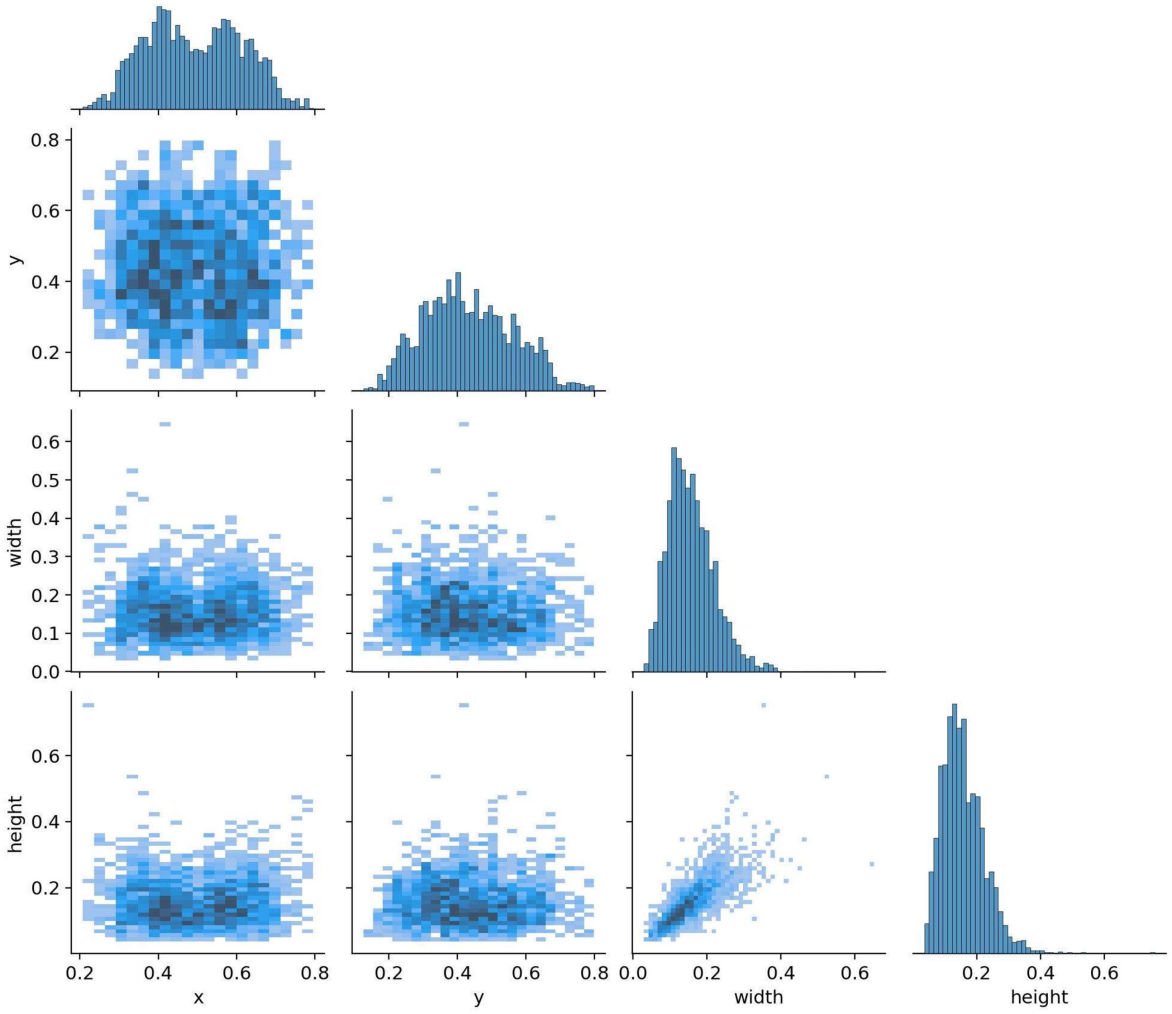
Target correlation graph in the dataset.

All images were pre-annotated by two radiologists with intermediate or higher professional titles using Labelimg for the segmentation targets in the images. The annotation results were reviewed on-site by two radiologists with deputy senior or higher professional titles to ensure the accuracy of the experiment. In this study, the model training was conducted using a five-fold cross-validation method. A random selection of 3,000 images from Dataset 1 was divided into five equally sized subsets, with each subset containing 600 images. These subsets were then split into training and validation sets in an 8:2 ratio, with four folds serving as the training set and the remaining fold as the validation set. This process was repeated five times, resulting in five distinct datasets for training. The remaining 223 images were used as an internal test set to evaluate the performance of the best model in segmenting targets. The annotation status in the training dataset and the correlation between different categories of targets are illustrated in Figures 8, 9.

### Experimental parameters

This experiment was conducted on a computer system equipped with a 13th Gen Intel (R) Core (TM) i7-13620H 2.40 GHz Central Processing Unit (CPU), NVIDIA GeForce RTX 4060 Laptop Graphics Processing Unit (GPU), and 16.0 GB Random Access Memory (RAM), using PyTorch to build the YOLOv5s model and YOLOv8s models (Python 3.8.7, PyTorch 2.0.0, CUDA 11.8). We trained seven distinct models, namely YOLOv5s, YOLOv5s-ASPP, YOLOv5s-CBAM, YOLOv5s-CA, YOLOv5s-ASPP-CBAM, YOLOv5s-ASPP-CA, and YOLOv8, on five datasets with varying data distributions. All models were optimized using the SGD optimizer, with a batch size of 8, an initial learning rate of 1 × 10^−2^, 100 training iterations, and an IOU threshold of 0.6.

### Experimental evaluation index

Referring to previous studies (Casas et al., 2024), unlike evaluation metrics for segmentation models such as U-net, the YOLOv5 segmentation model uses Precision, Recall, and mAP@50 to measure model performance. Precision refers to the proportion of samples predicted as True Positive (TP) out of all samples predicted as Lesions. It measures how many of the model’s predictions are truly correct. High precision indicates that the model has fewer False Positive (FP). Recall, on the other hand, refers to the proportion of actual TP that are correctly identified by the model. It emphasizes the model’s ability to find all true positives. High recall indicates that the model has fewer False Negative (FN). In the segmentation model task, *P* represents the proportion of predicted pixels that are correctly classified as the target, while R represents the proportion of actual target pixels that are correctly predicted as the target. The specific calculation formulas are shown in Equations 1, 2.


(1) $$\mathrm{Precision}=\frac{TP}{TP+FP}$$

(2) $$\mathrm{Recall}=\frac{TP}{TP+FN}$$

mAP@50 represents the average True Positive Rate (TPR) of the model when the precision reaches 50%. mAP@50 measures the proportion of predictions with an Intersection over Union (IoU) greater than or equal to 0.5, while the model is localizing the target and providing a confidence score.

A high mAP@50 indicates that the model can accurately identify targets with minimal positional prediction error. It is an important standard for measuring model performance, especially in real-time applications that focus on precision and efficiency.

Building on the aforementioned evaluation metrics, to better compare the computational capabilities of different models, we introduce the metric GFLOPs. GFLOPs is a common measure of the computational load of deep learning models. A higher GFLOPs value for a model indicates that it requires more computational resources and may necessitate more powerful hardware to support real-time or near-real-time inference.

The calculation formulas for mAP and AP are shown in Equations 3, 4, where *p* represents precision, r represents recall, and N represents the total number of sample categories. In this study, since it is a binary classification problem, the mAP value is the actual value of AP.


(3) $$\overset{1}{\underset{0}{\int }}p\left(r\right)dr$$

(4) $$\mathrm{mAP}=\frac{\sum AP}{N}$$

## Interpretation of result

**Figure 10:**
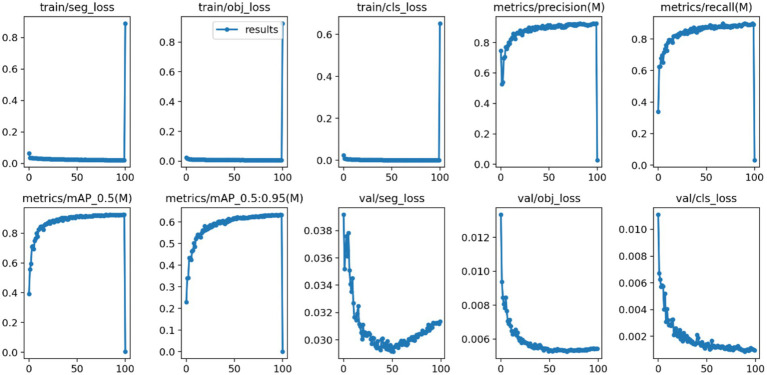
YOLOv5s model training curves.

**Figure 11:**
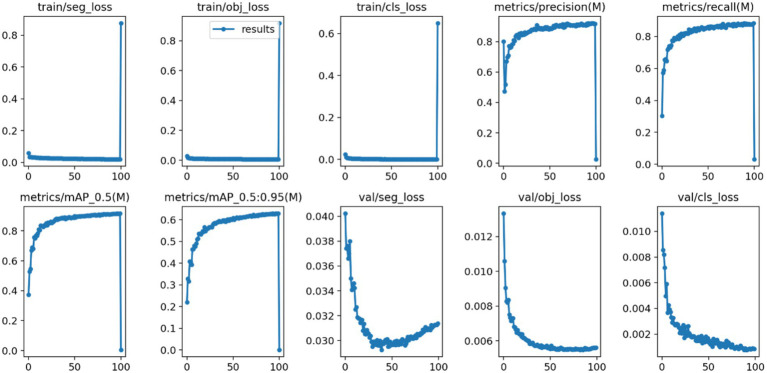
YOLOv5s-ASPP model training curves.

**Figure 12:**
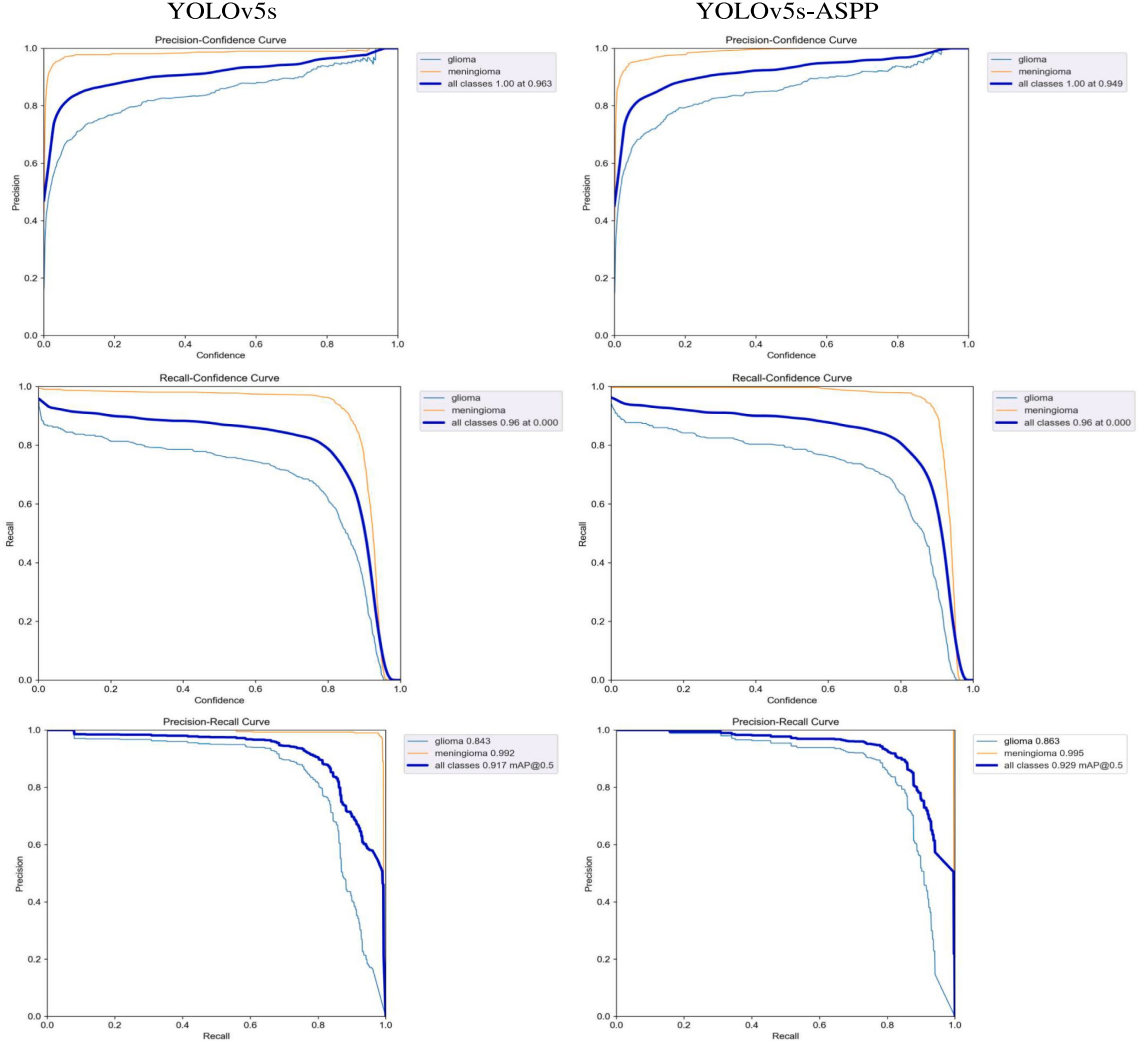
Model curve before and after improvement.

After 100 iterations of training, the precision, recall, mAP@50, and GFLOPs values of the six models on the validation set are shown in Table 1. The results of the model training curves are shown in Figures 10, 11. Figure 12 shows the precision curves, recall curves, and P-R curves for the YOLOv5s and YOLOv5s-ASPP models, with the area under the P-R curve representing mAP@50. Analyzing the table, we can see that the five improved models generally show improvements in precision, recall, mAP@50, and GFLOPs compared to the original YOLOv5s, with enhanced performance. Among them, the YOLOv5s-ASPP model exhibits significant improvements in all four evaluation metrics. At the same time, YOLOv8 is similar to YOLOv5-ASPP model in the precision, recall rate and mAP@50, but the GFLOPs of YOLOv8 is much larger than that of YOLOv5s-ASPP, which requires more computing resources.

**Table 1:** Comparison of values of evaluation indicators of YOLO model before and after improvement.

Name of model	*P*	*R*	mAP@50	GFLOPs
YOLOv5	0.928	0.868	0.917	25.7
YOLOv5s-ASPP	0.937	0.891	0.929	32.3
YOLOv5s-CBAM	0.903	0.884	0.913	26.0
YOLOv5s-CA	0.915	0.869	0.905	25.8
YOLOv5s-ASPP-CBAM	0.921	0.902	0.932	32.6
YOLOv5s-ASPP-CA	0.915	0.89	0.929	32.4
YOLOv8	0.931	0.919	0.945	42.4

**Figure 13:**
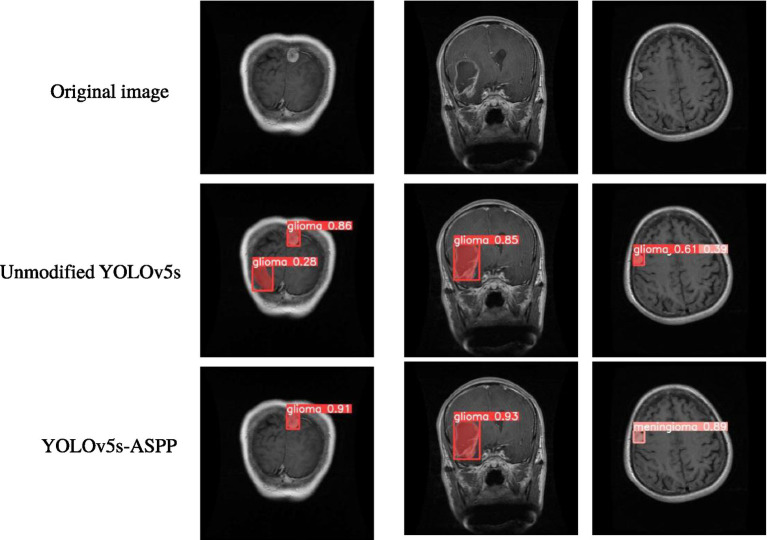
Comparison of YOLO model performance on the internal test set before and after improvement.

**Figure 14:**
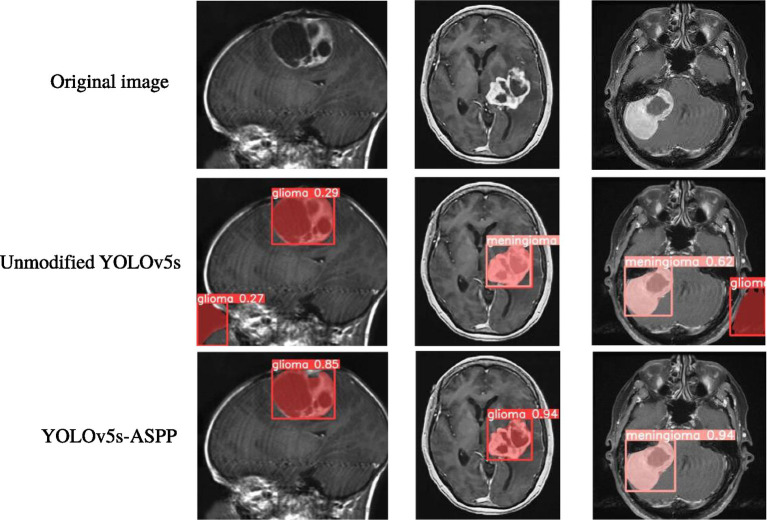
Segmentation results of YOLO models on the external test set before and after improvement.

The best-performing YOLOv5 and YOLOv5s-ASPP models from the five datasets were subjected to internal and external test sets to verify the detection results. The statistical outcomes are presented in Tables 2, 3, while the segmentation detection results for the internal and external test sets are illustrated in Figures 13, 14, respectively. The improved YOLOv5s model demonstrated superior segmentation and detection results for brain tumor MRI images.

**Table 2:** Comparison of YOLO model performance on the internal test set before and after improvement.

Name of model	*P*	*R*	mAP@50
YOLOv5	0.8	0.784	0.798
YOLOv5s-ASPP	0.854	0.784	0.826

**Table 3:** Comparison of YOLO model performance on the external test set before and after improvement.

Name of model	*P*	*R*	mAP@50
YOLOv5	0.809	0.688	0.741
YOLOv5s-ASPP	0.82	0.725	0.773

## Discussion

In this study, we introduced and validated an innovative variant of the YOLOv5s algorithm, which integrates attention mechanisms and the Atrous Spatial Pyramid Pooling (ASPP) structure. Our findings indicate that the incorporation of ASPP significantly bolsters the algorithm’s capacity for brain tumor segmentation. The enhanced model achieved a precision of 0.937, a recall of 0.891, and an mAP@50 score of 0.929. The mAP@50 score of 0.929 for the YOLOv5s-ASPP model is indicative of an exceptionally high level of accuracy in segmenting brain tumor MRI images, which is paramount for augmenting the precision and reliability of clinical diagnoses. This outcome highlights the substantial enhancement in segmentation capabilities conferred by the ASPP structure, which optimizes feature extraction and achieves high accuracy, rendering the YOLOv5s-ASPP model a valuable asset in clinical practice.

Brain tumor image segmentation is of significant value in clinical practice, as it can markedly enhance the precision of tumor diagnosis, aid in the formulation of personalized treatment plans, and is essential for monitoring treatment effects and assessing prognosis. Precise segmentation not only guides surgical path planning and reduces surgical risks but also serves for target delineation in radiotherapy planning, enabling a more accurate distinction between normal and tumor tissues. Consequently, this improves medical work efficiency and enhances patient education and treatment experiences. With advancements in technology, particularly in the realms of deep learning and artificial intelligence, the accuracy and automation level of brain tumor image segmentation are continually improving, heralding revolutionary changes in modern medicine.

In the domain of deep learning, various models, including CNNs and U-Net, have achieved success in brain tumor segmentation (Ding et al., 2020; Shen et al., 2023; Zhang et al., 2023). CNNs (Convolutional Neural Networks) excel in image processing and analysis but are characterized by high computational complexity, susceptibility to overfitting on small datasets, and sensitivity to hyperparameters. Furthermore, spatial resolution may be compromised during downsampling, impacting the capture of fine details (Sun and Pang, 2018). Conversely, U-Net, with its symmetrical architecture and skip connections, results in high memory consumption (Jeong et al., 2021), necessitates substantial computational resources, has a complex training process, and is prone to gradient propagation issues, posing challenges for practical clinical applications.

YOLOv5s has demonstrated significant superiority in the realm of real-time image detection. The YOLO model’s ability to directly predict the coordinates and positions of objects on the input image offers high generalization and transfer capabilities (Li et al., 2019). YOLOv5s is designed to balance speed and accuracy, providing satisfactory segmentation precision while maintaining high-speed detection. This makes YOLOv5s highly suitable for clinical diagnostics and treatment planning that demand rapid feedback. Moreover, the model’s less demanding computational resource requirements enable it to operate not only on CPUs but also to be deployed on devices with lower specifications, expanding its applicability across various environments.

Compared to its predecessor, the YOLOv5s model excels in real-time applications due to its swift detection speed and reduced computational costs. However, it struggles with segmenting small targets and complex backgrounds. This study introduces a structured improvement by incorporating the ASPP structure, leveraging its multi-scale context capturing capabilities to significantly enhance the model’s segmentation accuracy when dealing with tumors of varying sizes and morphologies. The refined YOLOv5s-ASPP exhibits notable improvements across multiple evaluation metrics, particularly with nearly 1–2% gains in precision, recall, and mAP@50, which is crucial for reducing misdiagnoses and missed diagnoses in clinical practice. Additionally, we explored four other models that integrate attention mechanisms. These models effectively enhance their generalization and data handling capabilities by amplifying essential image features and suppressing irrelevant ones. This improvement increases recall rates but slightly decreases precision. In brain tumor image segmentation tasks, a high recall rate ensures that tumor areas are not overlooked. Even with high precision, a low recall rate might lead to the neglect of some tumor parts. However, improving precision is equally vital, as it not only significantly boosts the accuracy of diagnostic segmentation, reducing the likelihood of misdiagnoses and missed diagnoses, but also optimizes treatment planning, ensuring effective tumor removal while maximizing the protection of brain functional areas and avoiding unnecessary damage.

However, this study still has its limitations. First, the data needs to be more diverse and multi-centric. Diverse and multi-centric data can enhance the model’s generalization ability and robustness. Therefore, further multi-centric studies on a larger scale are still needed. Second, the improved YOLOv5s still faces difficulties in accurately segmenting and detecting some tumors that are widely distributed, have large ranges, and unclear boundaries. Third, the lesion annotations in the dataset are all manually labeled, which is subjective and may contain errors.

YOLOv5 has shown excellent potential in the domain of medical image analysis. The model’s end-to-end training approach allows it to perform both object detection and segmentation tasks simultaneously, which is particularly important for improving the efficiency of medical image analysis. This technology will provide more favorable assistance to clinical practice.

Additionally, researchers may explore more deep learning techniques for use in brain functional imaging. Currently, deep learning has numerous applications in brain functional imaging. For instance, Zuo et al. proposed a model named BDHT, which utilizes generative adversarial networks to analyze multimodal brain networks for MCI, estimating effective connectivity and identifying potential biomarkers (Zuo et al., 2023, 2024a). The results indicate that this model surpasses existing methods in terms of accuracy and robustness, offering new insights into the diagnosis and treatment of MCI. Other researchers have developed the PALH model, a method that combines prior-guided adversarial learning with hypergraphs to predict abnormal brain connections in Alzheimer’s disease (Zuo et al., 2024b). This model demonstrates excellent performance in the analysis and prediction of AD progression and identifies abnormal connections that align with previous neuroscientific findings, which is significant for the study and early intervention of cognitive diseases. Experts have proposed the BSFL model, a brain structure–function representation learning framework that integrates DTI and fMRI data, aiming to enhance the analysis and prediction effects of MCI (Zuo et al., 2023, 2024a). This model outperforms other methods in predicting and analyzing MCI and has the potential to reconstruct a unified brain network and predict abnormal connections in the MCI process. Zong et al. (2024) proposed a paradigm based on diffusion graph contrastive learning (DGCL) for end-to-end construction of brain networks, enhancing the efficiency and generalization of brain disease analysis. DGCL outperforms traditional and deep learning methods in disease stage prediction, effectively identifying key brain connections and providing explanatory support for neurological diseases. There are many more application areas like the ones mentioned above that are worth exploring in the future, to assist clinical doctors in making more precise diagnoses and treatment plans, thereby improving patient prognosis and quality of life.

## Conclusion

In this paper, we propose an improved algorithm model based on YOLOv5, which undergoes a series of optimizations to the original YOLOv5 algorithm. By introducing the Atrous Spatial Pyramid Pooling (ASPP) structure, Convolutional block attention module, and Coordinate attention for efficient mobile network design, we have structurally improved the model and derived the following five optimized versions. After training under the same conditions as the original model and YOLOv8, we found that among the seven models, the improved YOLOv5s-ASPP model significantly enhanced the segmentation capability for brain tumor MRI images, aiding in diagnosis and treatment planning in clinical work.

## Data Availability

Publicly available datasets were analyzed in this study. This data can be found here: https://www.kaggle.com/datasets/masoudnickparvar/brain-tumor-mri-dataset/data; https://www.kaggle.com/datasets/sartajbhuvaji/brain-tumor-classification-mri.
